# Utilizing a large-scale biobanking registry to assess patient priorities and preferences for cancer research and education

**DOI:** 10.1371/journal.pone.0246686

**Published:** 2021-02-05

**Authors:** Cassandra A. Hathaway, Erin M. Siegel, Christine H. Chung, Smitha Pabbathi, Jennifer Vidrine, Susan Vadaparampil, Shelley S. Tworoger

**Affiliations:** 1 Department of Cancer Epidemiology, Moffitt Cancer Center, Tampa, Florida, United States of America; 2 Department of Head and Neck-Endocrine Oncology, Moffitt Cancer Center, Tampa, Florida, United States of America; 3 Department of Survivorship, Moffitt Cancer Center, Tampa, Florida, United States of America; 4 Department of Health Outcomes and Behavior, Moffitt Cancer Center, Tampa, Florida, United States of America; 5 Office of Community Outreach, Engagement, and Equity, Moffitt Cancer Center, Tampa, Florida, United States of America; 6 Department of Epidemiology, Harvard T.H. Chan School of Public Health, Boston, Massachusetts, United States of America; Nagoya University Asian Satellite Campuses Institute, Nagoya University Graduate School of Medicine, MYANMAR

## Abstract

Patients consented to biobanking studies typically do not specify research conducted on their samples and data. Our objective was to gauge cancer biobanking participant preferences on research topics. Patient-participants of a biobanking study at a comprehensive cancer center who had an appointment within the last 5 years, had a valid email address, and with a last known vital status of alive, were emailed a newsletter containing a link to a survey about preferences and priorities for research. The survey assessed demographics and research interest in three domains: cancer site, cancer-related topics, and issues faced by cancer patients. 37,384 participants were contacted through email to participate in the survey. 16,158 participants (43.2%) opened the email, 1,626 (4.3% overall, 10% of those who opened the email) completed the survey, and 1,291 (79.4% of those who completed the survey) selected at least one research priority. Among those who selected at least one research priorities for cancer-relevant topics, the most commonly selected were cancer treatment (66%), clinical trials (54%), and cancer prevention (53%). Similarly, the most selected priorities for cancer-related issues faced by patients were physical side effects of cancer (57%), talking to the oncologist (53%), and emotional challenges due to cancer (47%). Differences by gender were observed, with females reporting more interest in research generally. Cancer patients participating in a biobanking protocol prioritized research on treatments, prevention and side effects, which varied by gender.

## Introduction

Biobanks have become a cornerstone of medical research, particularly for complex diseases such as cancer [1, 2], with several notable new initiatives, including the Cancer Moonshot^SM^. The term biobank was first used in 1996 and since then, the number of articles mentioning biobanks has increased dramatically, many of which include cancer as the focus [2]. The number of articles published from biobanks has a yearly average growth rate above 7% [3]. Biobanks are a critical part of cancer research, and contribute to scientific collaboration, discovery of personalized treatments, and improved patient outcomes [2, 4, 5].

A unique feature of biobanks is that participants consent to broad use of biologic specimens and associated data. In this context, relatively little work has examined the patient perspective and priorities for use of the samples and data in research. Prior studies have examined patient and public preferences in relation to ethical issues in biobanking [6–9]; however, few have evaluated what type of research studies biobank participants value.

Cancer biobanks have the support of both health professionals [10] and patients, although consent rates have been found to vary by study and patient demographics [11, 12]. Nevertheless, limited research has been conducted to determine how to engage participants after consent. One qualitative study asked patients who consented to a biobank about communication, and 77% stated they would like to receive a newsletter [12]. Patients further stated that they were interested in research and would like to know the findings from the research conducted on their samples [12].

As a broader effort to engage participants of a large biobank within a comprehensive cancer center, a biannual newsletter was developed to foster enhanced bidirectional communication. The newsletter was designed to maintain engagement of participants through telling of participant stories and providing lay descriptions of research results conducted using the banked samples. Additionally, we incorporated a survey link asking about their priorities for self-education and cancer research. The aim of this study was to understand preferences for research topics among biobanking participants at a cancer center, and how those preferences varied by demographic factors, that could be used to guide research priorities of the biobank in the future.

## Material and methods

### Study population

Total Cancer Care (TCC), a large institutional prospective biobanking protocol (MCC#14690; Advarra, Inc. IRB Pro00014441), was initiated in 2006 at Moffitt Cancer Center. Patients consented to this protocol are 18 years of age or older, and agree to have their medical records, blood, tissue, and other biospecimens collected for research. A key feature to TCC is that patients also agree to be followed for life through registry linkage, give permission to be re-contacted if they are found to be eligible for a clinical trial or other research study, and agree explicitly agree that their data and biospecimens will be shared with internal and external investigators. Because participants had already provided informed written consent to the TCC protocol, which includes being re-contacted to complete the questionnaire, we obtained a waiver of consent for this study, although the electronic newsletter and questionnaire, described below, were approved by the IRB. At the time of this study on January 31, 2020, 117,528 Moffitt Cancer Center patients had consented to the TCC protocol. The participants include patients of all cancer types as well as individuals without cancer who may have come to the cancer center for other reasons (e.g., screening, genetic risk assessment), though the majority of the population has had a diagnosis of cancer (~98%).

Participants in this study were selected from among TCC consented patients who had been seen at Moffitt Cancer Center anytime since January 1, 2015, had a last known vital status of alive, and had a valid email address (n = 37,384). Eligible individuals received an electronic letter or “eLetter” email from the TCC principal investigator, the first of a series of biannual eLetters, thanking the participants for their contribution to research, highlighting research that derived from the biobank, and describing key elements of the protocol. This eLetter is a recently introduced initiative designed to engage TCC-enrolled patients and inform them of activities and research being conducted as a result of their participation. At the end of the initial article in the eLetter, a link was included to complete an anonymous survey regarding topics participants might be interested in learning about and research areas of importance. The subject line of the email did not indicate that a survey was embedded in the eLetter. Further, the email was sent only once with no follow-up reminders to complete the survey. The email was sent on January 31^st^, 2020 and the survey was closed to data collection on March 12^th^, 2020.

### Data collection

The survey contained questions on demographics, including age, gender, ethnicity, race, and county of residence (full survey available in S1 File). Participants were asked to provide input on topics of potential research interest across three domains: cancer site (n = 14 items), cancer-related topics (n = 10 items), and issues faced by cancer patients (n = 12 items). Participants selected all topics of interest in each domain and were provided an opportunity for free text responses. Questions were “Please indicate your interest based on the following top cancers in our catchment area,” “Please indicate your interest based on the following cancer-related topics,” and “Please indicate your interest based on the following issues often faced by cancer patients.” Each of the main questions had two sub-questions to gauge personal interest in learning more about that item and desire to have research conducted in that area by the cancer center.

Cancer-related topics included tobacco cessation, cancer prevention, cancer screening, cancer treatment, cancer survivorship, cancer clinical trials, nutrition and cancer, genetics and cancer, biobanking and cancer, and cancer caregiving. Issues faced by cancer patients included housing, transportation, child care, and job; insurance issues; talking to the oncologist; talking to the primary physician; talking to their friends/family; care-takers of cancer patients; emotional challenges due to cancer; memory and concentration problems; physical side effects of cancer; fatigue and poor sleep; diet and exercise; and fertility options after cancer. These questions were adapted from the Clinical Needs Assessment Tool for Cancer Survivors [13]. Cancer sites were based on the top cancers in our catchment area and included breast, lung, melanoma, prostate, colorectal, bladder, head and neck, Non-Hodgkin lymphoma, kidney, brain tumors, thyroid, uterine, ovarian, cervical and a write in option.

### Statistical analysis

For each response in the three domains, we calculated the percent of people who selected the item out of the total number of people who responded to that question for both interest in learning about the topic and interest in having research conducted on that topic. Chi-square tests were performed to compare differences by gender (male/female), age (18-59/≥60), race/ethnicity (non-Hispanic white/Hispanic or non-white), and geographical area (inside catchment area/outside catchment area). The catchment area for Moffitt Cancer Center consists of a 15-county region in West Central Florida in which 71% of Moffitt Cancer Center patients live. Cancer sites were grouped according to the SEER site groups [14] (e.g. GYN consists of ovarian, cervical, and uterine cancers). All *P-*values were 2-sided and were considered statistically significant if less than 0.05. Analyses were conducted using SAS, version 9.4 (SAS Institute Inc., Cary, North Carolina, United States).

## Results

In total, 37,384 TCC-consented Moffitt patients were emailed the eLetter, 16,158 of those (43.2%) opened the email, and 1,626 (4.3%) completed the survey; 2,299 emails were undeliverable. Compared to all those who received the email, those who responded to the survey were slightly older and more likely to be female, white, and not Hispanic or Latinx (Table 1). Respondents to the survey were more likely to live in the Moffitt’s catchment area versus outside the catchment area (71.1% versus 28.9%, respectively), however, compared to those who received the email, respondents were less likely to be from the catchment area (74.3% versus 71.1%, respectively). The geographical distribution is consistent with the distribution of new patients Moffitt sees in their catchment area each year and those who enroll in TCC (71%).

**Table 1:** Characteristics of participants in a cancer-related biobanking study who received an eLetter, those who opened the email and those who responded to an embedded survey at the end of the first article.

	Recipients ^b^ (n = 37,384)		Opened Email ^b^ (n = 16,158)		Respondents (n = 1,626)	
	n	%^a^	n	% ^a^	n	% ^a^
**Age**						
18–39	1845	4.9%	643	4.0%	29	1.8%
40–44	1224	3.3%	430	2.7%	23	1.5%
45–49	1758	4.7%	649	4.1%	40	2.5%
50–54	2676	7.2%	1038	6.5%	69	4.4%
55–59	3988	10.7%	1585	9.9%	160	10.1%
60–64	4803	12.9%	2079	13.0%	206	13.0%
65–69	5632	15.1%	2609	16.3%	320	20.2%
70–74	6233	16.7%	2999	18.8%	372	23.5%
75–79	5025	13.5%	2291	14.3%	227	14.3%
80 or over	4150	11.1%	1671	10.5%	137	8.7%
Missing ^c^	50		164		43	
**Gender**						
Female	20291	54.3%	8463	52.8%	894	55.9%
Male	17092	45.7%	7553	47.2%	695	43.5%
Prefer not to answer	0	0.0%	0	0.0%	9	0.6%
Missing ^c^	1		142		28	
**Ethnicity**						
Hispanic or Latinx	2611	7.1%	1042	6.6%	74	4.7%
Not Hispanic or Latinx	34378	92.9%	14788	93.4%	1499	95.3%
Missing ^c^	395		328		53	
**Race**						
American Indian or Alaska Native	60	0.2%	26	0.2%	7	0.4%
Asian	432	1.2%	191	1.2%	7	0.4%
Black or African American	1755	4.7%	615	3.9%	37	2.3%
Native Hawaiian or Other Pacific Islander	40	0.1%	19	0.1%	1	0.1%
White	33682	90.4%	14564	91.2%	1478	92.8%
Prefer Not to Say	155	0.4%	63	0.4%	29	1.8%
Other (not specified)	965	2.6%	400	2.5%	14	0.9%
Mixed	158	0.4%	89	0.6%	20	1.3%
Missing ^c^	137		191		33	
**Geographical Area**						
Inside Catchment Area	27767	74.3%	11682	73.1%	1115	71.1%
Outside Catchment Area	9607	25.7%	4297	26.9%	454	28.9%
Missing ^c^	10		179		57	

^a^ Percentages are based on non-missing values

^b^ Data for total email recipients and those who opened the email came from medical records

^c^ Missing values may differ between groups due to email addresses not aligning with medical records

**Fig 1:**
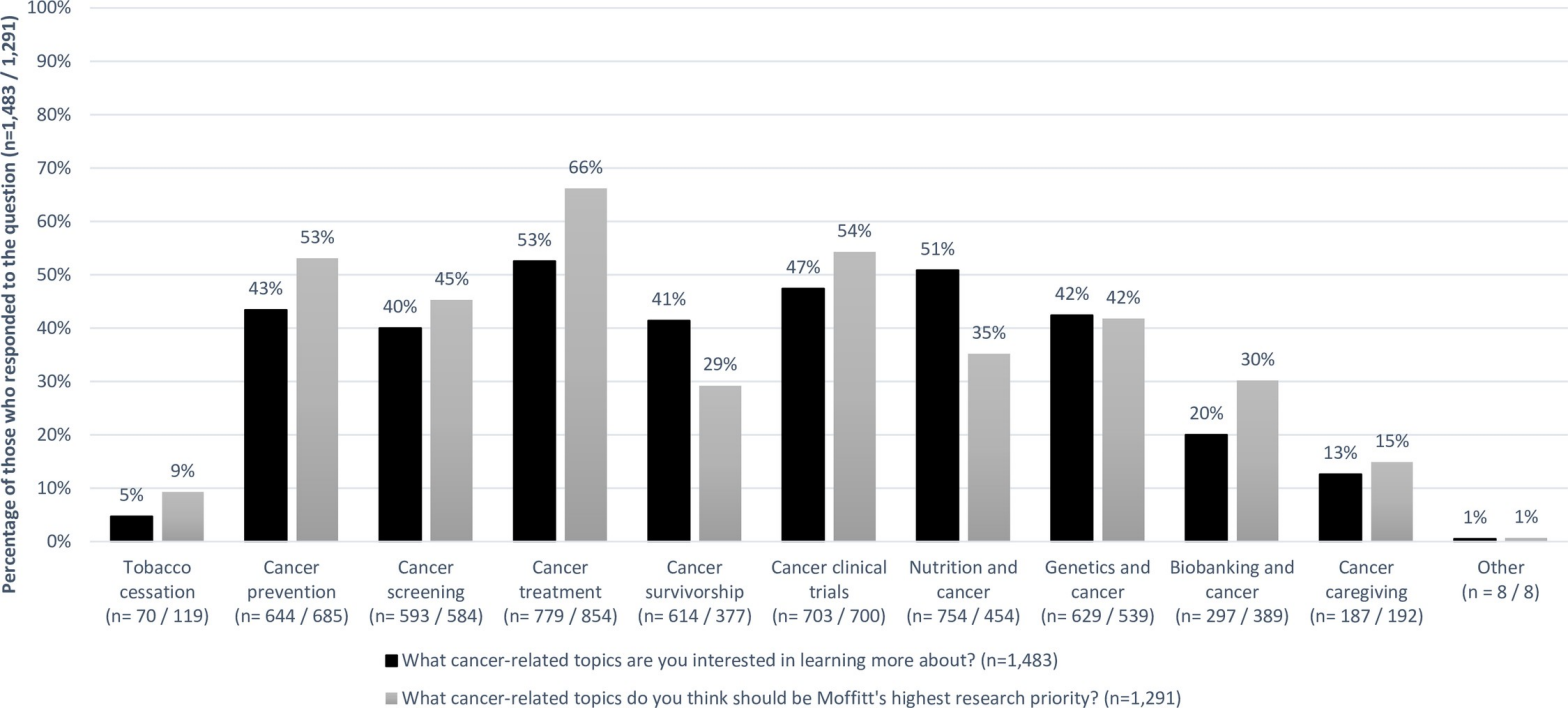
Percentage of respondents who selected having an interest in learning about and having research conducted on multiple cancer-related topics. Black bars: percentage of respondents who selected cancer-related topic having an interest in learning more about, out of total number of people who responded to the question (n = 1,483). Gray bars: percentage of respondents who selected cancer-related topic having research conducted on, out of total number of people who responded to the question (n = 1,291).

**Fig 2:**
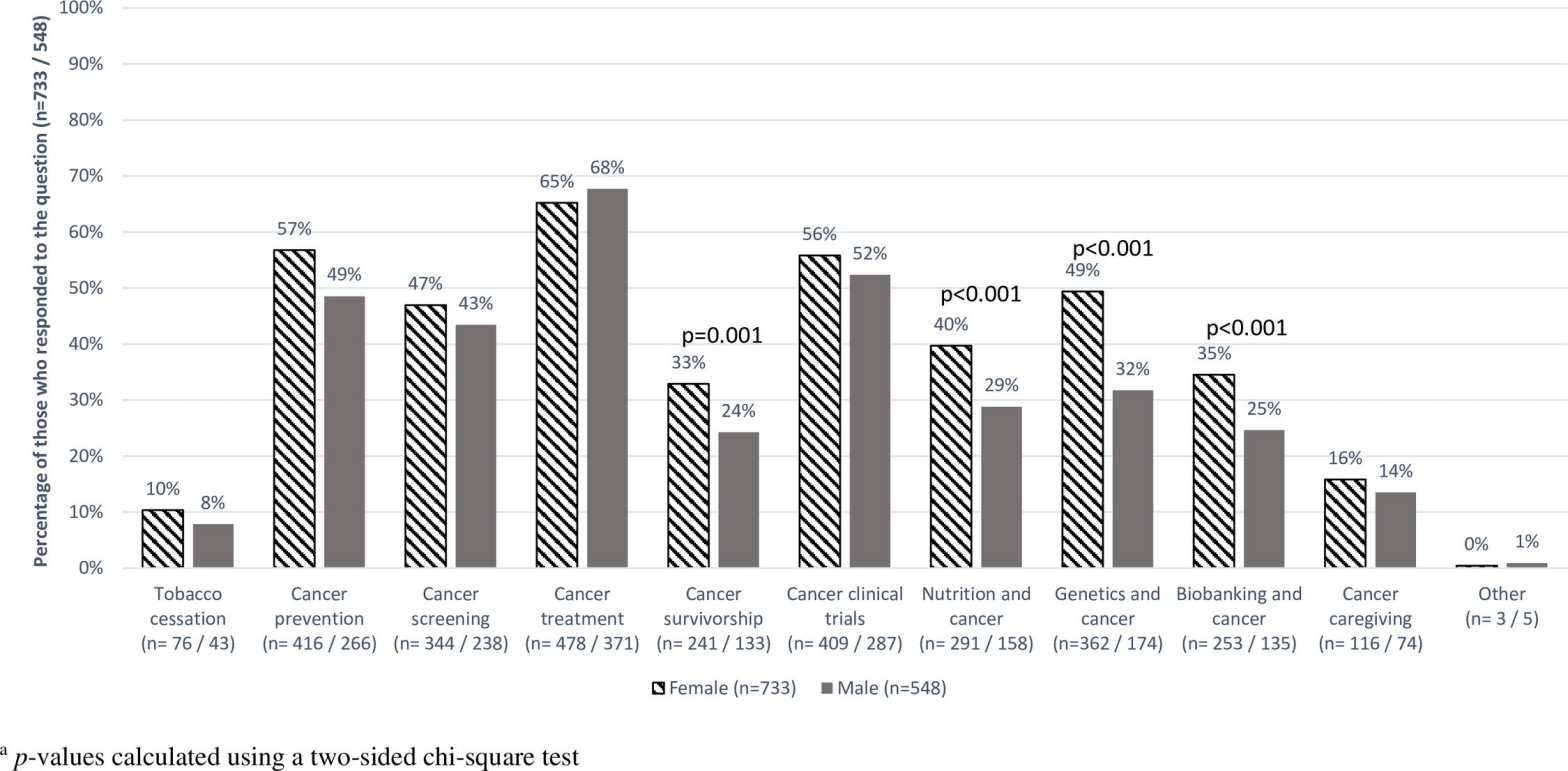
Percentage of respondents who selected having research conducted on multiple cancer-related topics by gender. Horizontal striped bars: percentage of females who selected cancer-related topic having research conducted on, out of total number of females who responded to the question (n = 733). Dark gray bars: percentage of males who selected cancer-related topic having research conducted on, out of total number of males who responded to the question (n = 548).

For cancer-related topics, 1,483 (91.2%) respondents selected at least one topic of interest to learn about and 1,291 (79.4%) selected a research topic of interest. The top four areas selected by participants to learn about were cancer treatment (53%), nutrition (51%), clinical trials (47%), and cancer prevention (43%) (Fig 1). Three of these overlapped with the most commonly selected research priorities (cancer treatment, 66%; clinical trials, 54%; cancer prevention, 53%); cancer screening (45%) was also of research interest. Females versus males were more likely to choose cancer prevention (57% vs. 49%, p = 0.004), cancer survivorship (33% vs. 24%, p = 0.001), nutrition (40% vs. 29%, p<0.001), genetics (49% vs. 32%, p<0.001), and biobanking (35% vs. 25%, p<0.001) as research priorities (Fig 2). Nutrition was largely of personal interest to learn about, with 26% of participants selecting it to learn more about, 6% selecting it as a research priority, and 23% selecting both (S1 Table). Those who selected nutrition as a research priority were more likely to be younger (<60 years old), and Hispanic or Non-White (S2 Table). Younger and Hispanic or Non-White participants were also more likely to choose cancer caregiving as a research priority, and younger participants were more likely to choose cancer treatment and genetics as research priorities. Results for cancer-related topics were similar by geographical area.

**Fig 3:**
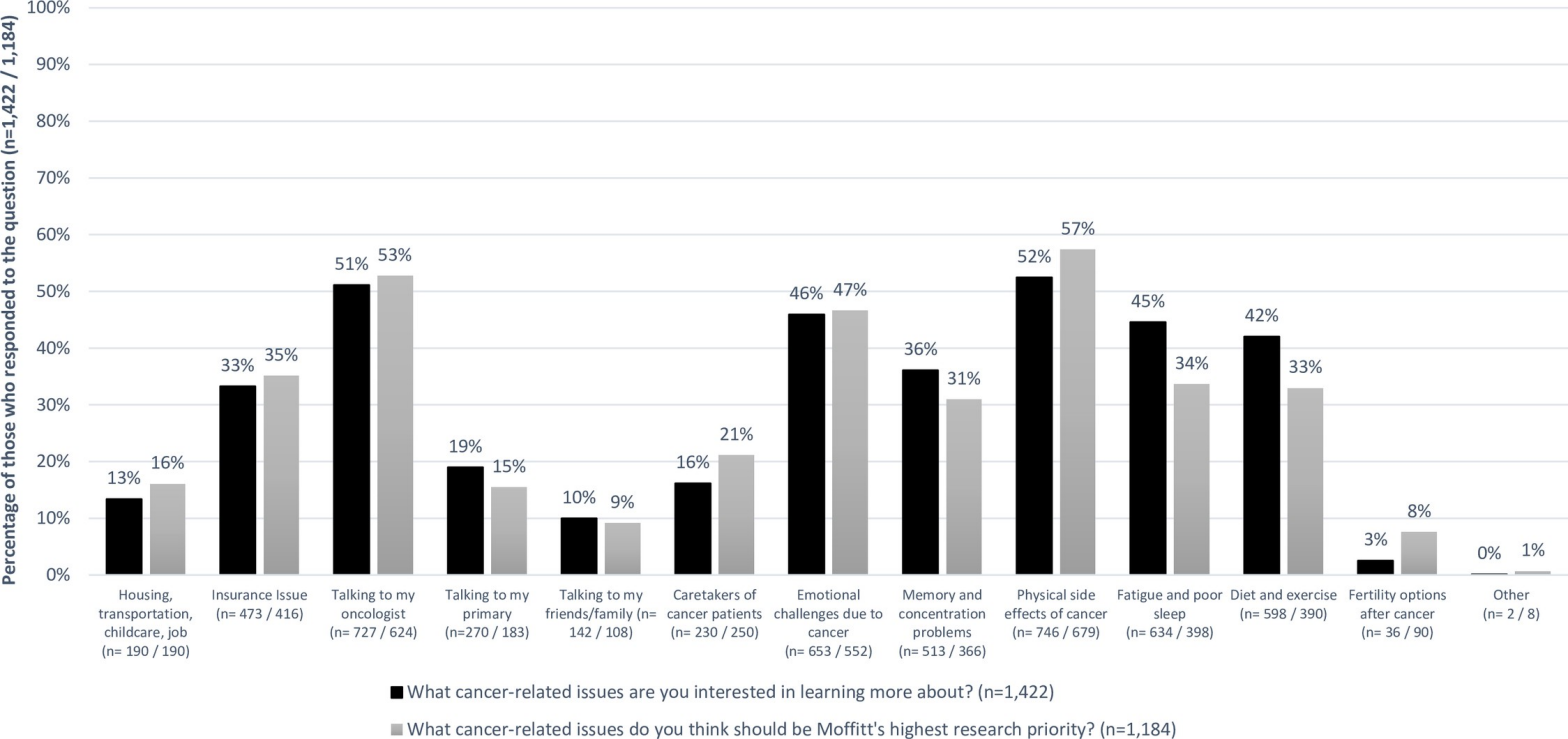
Percentage of respondents who selected having an interest in learning about and having research conducted on multiple cancer-related issues. Black bars: percentage of respondents who selected cancer-related issues having an interest in learning more about, out of total number of people who responded to the question (n = 1,422). Gray bars: percentage of respondents who selected cancer-related issues having research conducted on, out of total number of people who responded to the question (n = 1,184).

**Fig 4:**
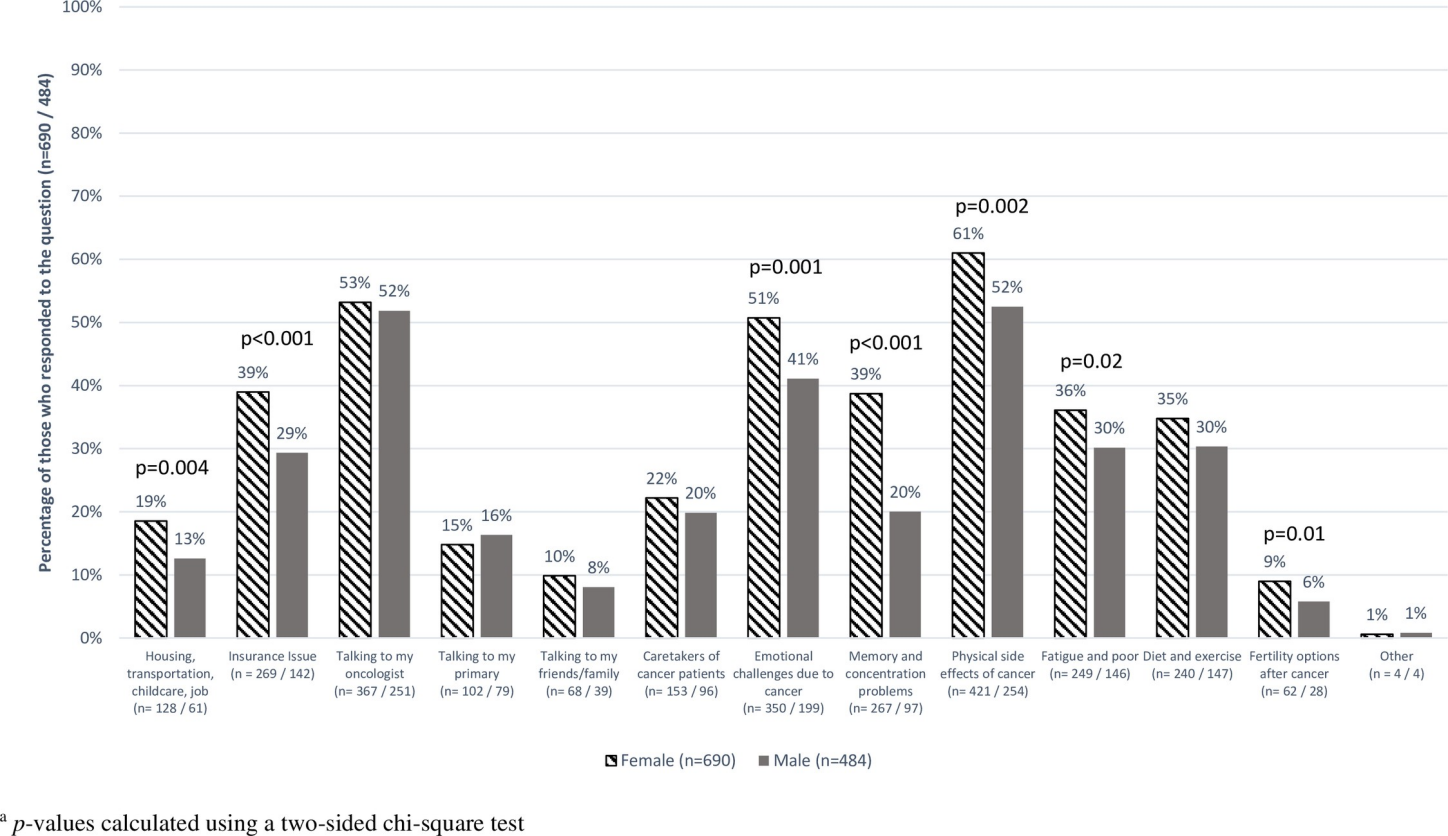
Percentage of respondents who selected having research conducted on multiple cancer-related issues, by gender. Horizontal striped bars: percentage of females who selected cancer-related issues having research conducted on, out of total number of females who responded to the question (n = 690). Dark gray bars: percentage of males who selected cancer-related issues having research conducted on, out of total number of males who responded to the question (n = 484).

Regarding cancer-related issues, 1,422 (87.4%) chose at least one topic of interest to learn about and 1,184 (72.8%) participants selected a research topic. Approximately half were interested in learning more about the physical side effects of cancer (52%), talking to their oncologist (51%), and the emotional challenges due to cancer (46%) (Fig 3). Research interests were similar (57%, 53%, and 47%, respectively). Research priorities by gender varied, with females communicating more interest than males regarding housing, transportation, childcare, and job (19% vs. 13%, p = 0.004); insurance (39% vs. 29%, p<0.001); emotional challenges due to cancer (51% vs. 41%, p = 0.001); memory and concentration problems (39% vs. 20%, p<0.001); physical side effects of cancer (61% vs. 52%, p = 0.002); fatigue and poor sleep (36% vs. 30%, p = 0.02); and fertility options after cancer (9% vs. 6%, p = 0.01) (Fig 4). Hispanic or Non-White participants were more likely to select housing, transportation, childcare and job as well as diet and exercise as research priorities, than Non-Hispanic white participants (S3 Table). Younger participants also selected insurance issues, memory and concentration problems, fatigue and poor sleep, and fertility options after cancer as research priorities more so than those over 60 years old. Research priorities for cancer-related issues were similar by geographical area.

Among 1,357 (83.4%) respondents, the top three cancer sites patients wanted to learn about were genitourinary (GU) (41%), breast (36%), and skin (33%) cancers. Breast and GU cancers were also among the top three cancer sites patients thought should be research priorities (57% and 46%, respectively), as well as brain cancer (44%) 1,077 (66.2%) people chose a cancer site research area of interest. For all cancer sites except for GU and skin, more patients selected that the cancer should be a research priority than selected that they were interested in learning about that cancer (S1 Fig; S3 Table).

## Discussion

In a brief survey of a large cohort of patients who were enrolled in a cancer biobanking study, we observed that patients prioritized research on cancer treatments, including clinical trials, as well as cancer prevention, physical and emotional side effects of cancer and talking to their oncologist. These largely aligned with topics that patients wanted to learn more about personally. While priority selections did not differ substantially by age, race/ethnicity, or within the catchment area of the cancer center versus not, females tended to be more likely to complete the survey overall and to select more topics as important for research compared to males, particularly biobanking, survivorship issues, and practical challenges faced by cancer patients, such as insurance.

Our study demonstrates that cancer patients have specific preferences about important areas of research as well as receiving communication and education regarding research conducted within a biobank. While 16.4% of individuals who completed our survey did not select any research topic of interest, the vast majority selected at least one topic (S2 Fig). Many of the participant-selected research interests were in domains that are traditional strengths of biobanks, such as development of treatments or treatment targets, early detection, and informing clinical trials. However, the patients in our study expressed a desire for more research about emotional consequences of cancer, patient-provider communications, and other non-biomarker-based research topics. This is consistent with a qualitative analysis of cancer patients in the United Kingdom in which patient priorities for research included the impact of cancer on one’s life and related support issues; risk factors and causes; early detection and prevention; and treatments [15]. Notably, oncology patient interests for research are consistent with recommendations by a Lancet Oncology Commission for cancer research priorities in the US to include precision cancer prevention, drug discovery and development, immunotherapy, and supportive oncology [16]. It is interesting to note that in both the UK and our study, patients in a cancer biobank valued conducting research on factors such as prevention and quality of life issues in a similar proportion to developing novel treatments. This suggests that biobanks could extend research into areas such as patient-reported outcomes and shared decision-making across the cancer continuum as participants may be willing to provide additional data (e.g., questionnaires) to support such research [17].

Similar to prior studies [12, 18–20], patients in our study were interested in learning both about their disease and the research generated from the biobank in topic areas that spanned from prevention to early detection to survivorship. Our findings suggest that electronic communications from cancer-related biobanks should cover a variety of topics across the cancer continuum and focus on areas of interest to the participants as this may increase patient engagement in biobanking research and potentially address educational gaps experienced by participants. However, most biobanks do not share information about the associated research [18, 19]. Reasons cited for not sharing results included concerns with participant health literacy, logistical barriers, and a lack of desire for results [21]. Our study demonstrated that many participants want to understand more about research results as well as have distinct opinions about what type of research is important to conduct.

In our study, women were more likely than men to select at least one research priority (82% vs. 79% for cancer-related topics and 77% vs. 70% for cancer-related issues, respectively). The specific topics of higher interest to women compared to men encompassed financial/ practical challenges (e.g., housing, childcare, insurance) as well as psychosocial (e.g., emotional challenges, memory problems, fatigue) and physical side effects of cancer, including fertility. Our findings are consistent with a qualitative study of 12 female cancer patients, who noted that everyday activities and coordination as well as work to maintain income were substantially impacted by cancer [22], and another study showing that breast cancer patients loss of work may impact insurance coverage [23]. In addition, a longitudinal study of 237 breast cancer patients demonstrated that impacts of lost income, health expenses, and lost unpaid work lasted at least 18 months [24]. Further, it has been shown women with cancer experience have more anxiety and depression than men [25, 26] as well as more severe side effects from cancer treatments [27], supporting their interest in research to abrogate these issues. These findings may be attributed to differences in cancer diagnosis between men and women and further research is needed to better understand preferences by gender, cancer type, and how that may lead to targeted communication for women versus men in biobank settings.

This study includes many strengths, one of which is the large sample size in an established cancer biobank, with over 36,000 patients sent an eLetter about the biobank study, who represent a diverse range of cancer types. Given that it was the first communication of its kind in this biobank, a high proportion of individuals opened the email. However, a limitation is the link to the survey was embedded at the end of the first article, such that even among those who opened the email, some may not have seen the survey link. This may explain the relatively low response rate (10% of all those who opened the email) and lead to bias in the results. Responses could be improved if the primary email was about the survey and reminder emails were sent. Respondents were less likely to be Hispanic and non-white which may be due to the survey being electronic and only given in English [28], as well as the lack of an incentive. Additionally, we did not include any patient identifiers in the survey so there was no way to link the data back to the patients’ medical records; this was done to maintain anonymity and foster honest feedback of the participants. Future studies should gather more detail regarding cancer type and potentially linking survey responses with medical records to understand differences by cancer diagnosis as well as better understand reasons for participating in the biobank. Future studies may also want to consider formatting questions using a rank-based approach to understand the relative importance of different research opportunities rather than mark all that apply. While this study was conducted at a single site, there are many similar cancer biobanks that could conduct similar research to expand understanding across different populations.

Overall, we found that participants in a cancer biobank are interested in learning about and having research conducted on a wide range of topics that span the cancer continuum, from prevention to survivorship and vary by demographics. This information can be used to improve communication between researchers and participants, increasing both satisfaction and engagement and potentially improving participation in future research activities, such as longitudinal surveys or additional sample collections. This may also inform governance of biobank by integrating participant interest with how data and samples are dispersed. This is consistent with proposed approaches for ensuring ethically responsible use of samples from biobanks utilizing broad-based consents [29]. Lastly, as demonstrated by the majority of people who selected at least one research priority or one area of personal interest, participants in a cancer biobank are interested in learning more from the research conducted on their samples, and there is a need to overcome the disconnect between patient preferences for obtaining research results and the researchers’ expectations of distributing results, as well as improve communication of research findings with patients, and developing bi-directional approaches that align research and patient needs.

## Supporting information

S1 Fig Percentage of respondents who selected having an interest in learning about and having research conducted on multiple cancer sites. Black bars: percentage of respondents who selected cancer sites having an interest in learning more about, out of total number of people who responded to the question (n = 1,357). Gray bars: percentage of respondents who selected cancer sites having research conducted on, out of total number of people who responded to the question (n = 1,077).
(TIF)

S2 Fig Number of research topics each participant selected overall (n = 1,626). Gray bars: number of research topics selected by participants overall for 32 items across three domains, plus three write in options (one per domain).
(TIF)

S1 Table Number and percentage of respondents who selected having only a personal interest in the topic, only an interest to have research conducted on the topic, or both. (DOCX)

S2 Table Percentage of respondents who selected having research conducted on cancer-related topics by demographic characteristics, including age, race/ethnicity, and geographic location. (DOCX)

S3 Table Percentage of respondents who selected having research conducted on multiple cancer-related issues by demographic characteristics, including age, race/ethnicity, and geographic location. (DOCX)

S1 File Survey. (PDF)
