# Supplementary figures and images for: Utilizing a large-scale biobanking registry to assess patient priorities and preferences for cancer research and education
Source: PLoS One. 2021 Feb 5;16(2):e0246686. doi: 10.1371/journal.pone.0246686 (PMC7864448; doi:10.1371/journal.pone.0246686)

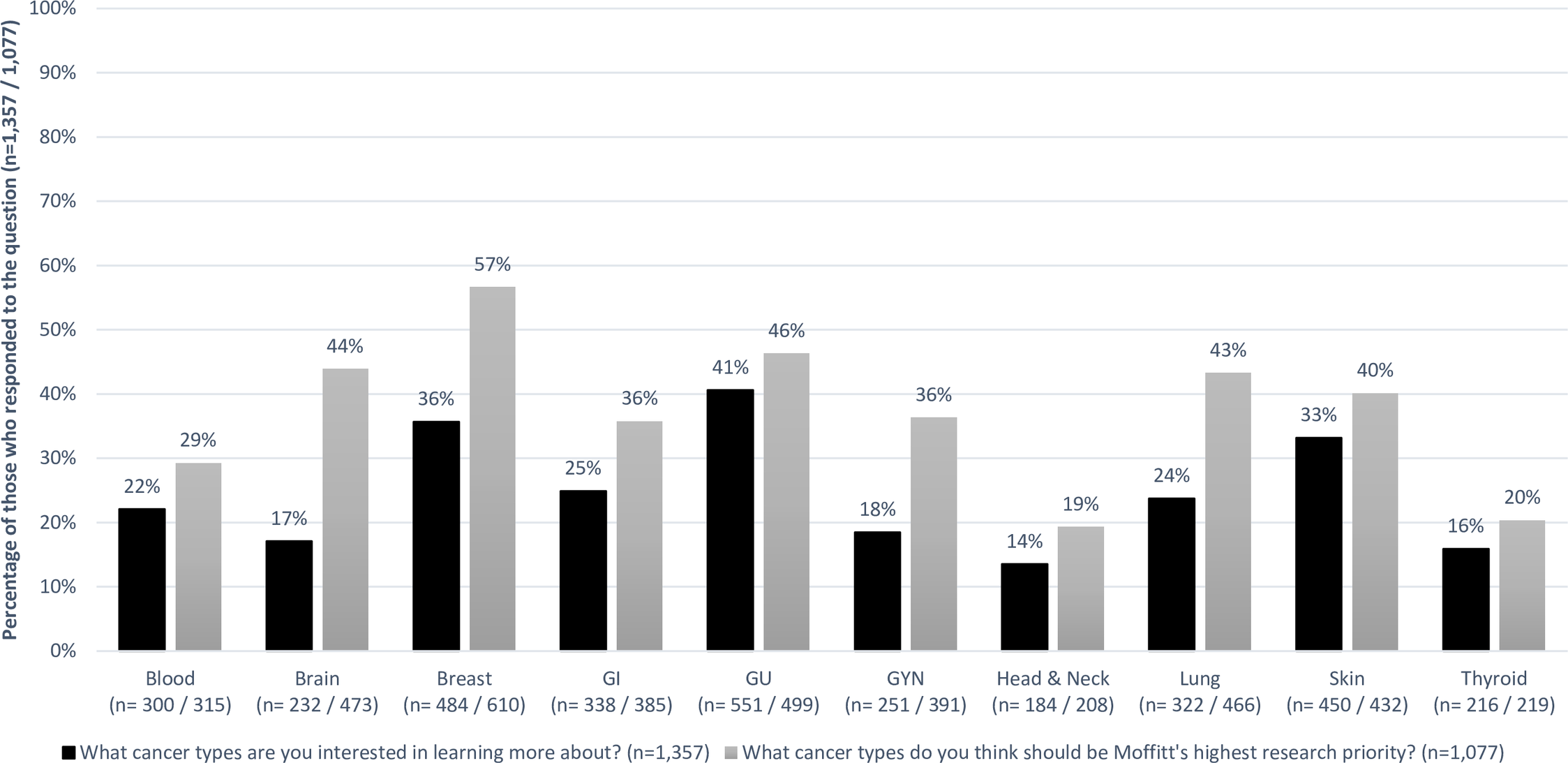

Supplement: S1 Fig — Black bars: percentage of respondents who selected cancer sites having an interest in learning more about, out of total number of people who responded to the question (n = 1,357). Gray bars: percentage of respondents who selected cancer sites having research conducted on, out of total number of people who responded to the question (n = 1,077). (TIF) [file pone.0246686.s001.tif]

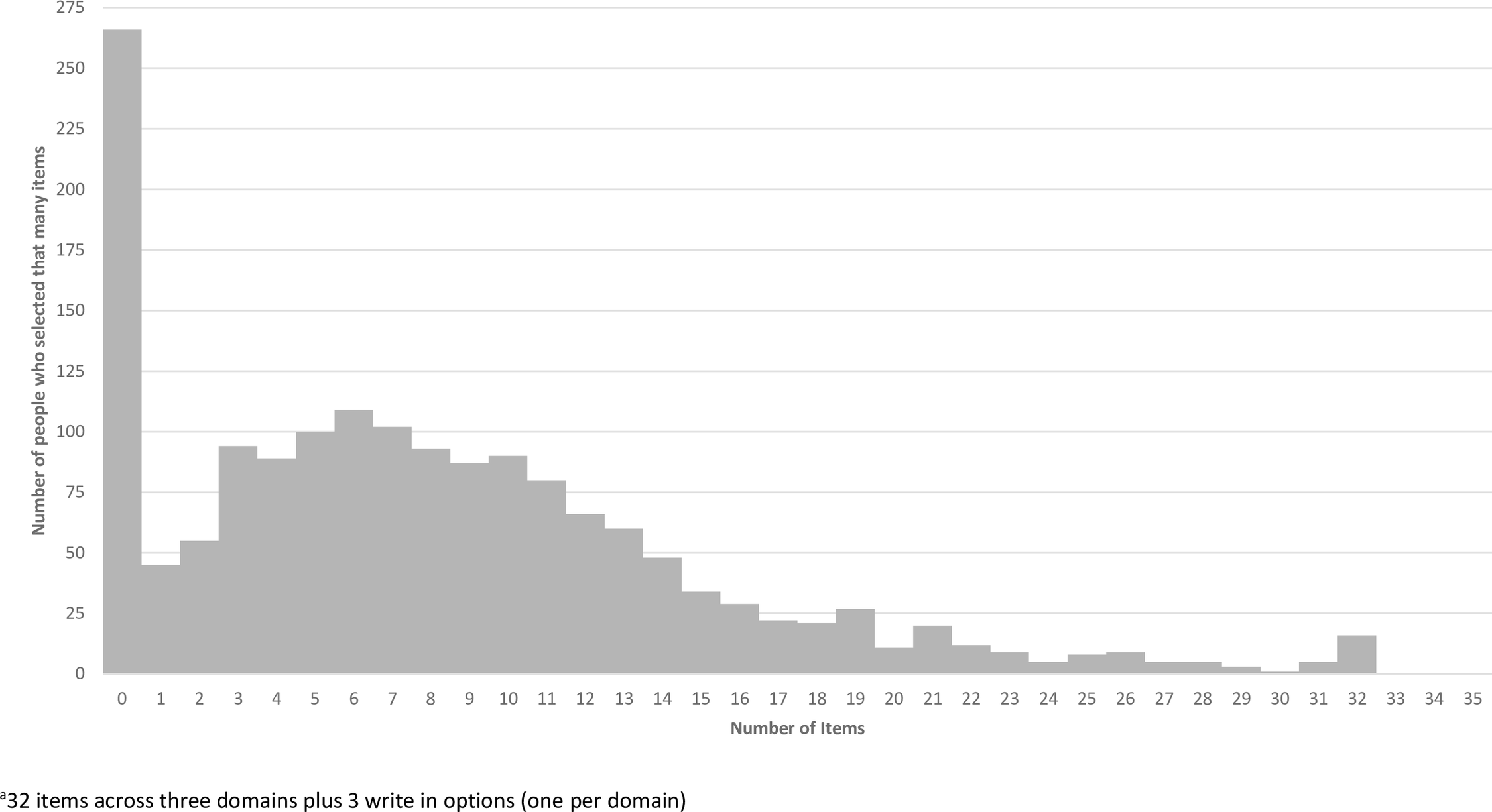

Supplement: S2 Fig — Gray bars: number of research topics selected by participants overall for 32 items across three domains, plus three write in options (one per domain). (TIF) [file pone.0246686.s002.tif]
