# Supplementary material for: Utilizing a large-scale biobanking registry to assess patient priorities and preferences for cancer research and education
Source: PLoS One. 2021 Feb 5;16(2):e0246686. doi: 10.1371/journal.pone.0246686 (PMC7864448; doi:10.1371/journal.pone.0246686)
Supplement: S1 Table — (DOCX) [file pone.0246686.s003.docx]

**S1 Table.** **Number and percentage of respondents who selected having only a personal interest in the topic, only an interest to have research conducted on the topic, or both.**

| **Cancer-Related Topics (n=1552)** | | | |
| --- | --- | --- | --- |
|  | **Number (%^a^) who were only interested in learning more about the topic** | **Number (%^a^) who thought the topic should only be a research priority** | **Number (%^a^) who wanted to learn more AND thought it was a research priority** |
| Tobacco cessation | 44 (3%) | 93 (6%) | 26 (2%) |
| Cancer prevention | 221 (14%) | 262 (17%) | 423 (27%) |
| Cancer screening | 244 (16%) | 235 (15%) | 349 (22%) |
| Cancer treatment | 225 (14%) | 300 (19%) | 554 (36%) |
| Cancer survivorship | 353 (23%) | 116 (7%) | 261 (17%) |
| Cancer clinical trials | 246 (16%) | 243 (16%) | 457 (29%) |
| Nutrition and cancer | 399 (26%) | 99 (6%) | 355 (23%) |
| Genetics and cancer | 258 (17%) | 168 (11%) | 371 (24%) |
| Biobanking and cancer | 112 (7%) | 204 (13%) | 185 (12%) |
| Cancer caregiving | 105 (7%) | 110 (7%) | 82 (5%) |
| **Cancer-Related Issues (n=1504)** | | | |
| Housing, transportation, childcare, job | 115 (8%) | 115 (8%) | 75 (5%) |
| Insurance Issue | 220 (15%) | 163 (11%) | 253 (17%) |
| Talking to my oncologist | 271 (18%) | 168 (11%) | 456 (30%) |
| Talking to my primary | 163 (11%) | 76 (5%) | 107 (7%) |
| Talking to my friends/family | 97 (6%) | 63 (4%) | 45 (3%) |
| Caretakers of cancer patients | 113 (8%) | 133 (9%) | 117 (8%) |
| Emotional challenges due to cancer | 280 (19%) | 179 (12%) | 373 (25%) |
| Memory and concentration problems | 249 (17%) | 102 (7%) | 264 (18%) |
| Physical side effects of cancer | 271 (18%) | 204 (14%) | 475 (32%) |
| Fatigue and poor sleep | 332 (22%) | 96 (6%) | 302 (20%) |
| Diet and exercise | 295 (20%) | 87 (6%) | 303 (20%) |
| Fertility options after cancer | 11 (1%) | 65 (4%) | 25 (2%) |
| **Cancer Sites (n=1463)** | | | |
| Blood | 140 (10%) | 155 (11%) | 160 (11%) |
| Brain | 71 (5%) | 312 (21%) | 161 (11%) |
| Breast | 152 (10%) | 278 (19%) | 332 (23%) |
| GI | 163 (11%) | 210 (14%) | 175 (12%) |
| GU | 248 (17%) | 196 (13%) | 303 (21%) |
| GYN | 90 (6%) | 230 (16%) | 161 (11%) |
| Head & Neck | 100 (7%) | 124 (8%) | 84 (6%) |
| Lung | 114 (8%) | 258 (18%) | 208 (14%) |
| Skin | 193 (13%) | 175 (12%) | 257 (18%) |
| Thyroid | 116 (8%) | 119 (8%) | 100 (7%) |

^a^ percentages calculated from total n
