# Supplementary material for: Utilizing a large-scale biobanking registry to assess patient priorities and preferences for cancer research and education
Source: PLoS One. 2021 Feb 5;16(2):e0246686. doi: 10.1371/journal.pone.0246686 (PMC7864448; doi:10.1371/journal.pone.0246686)
Supplement: S2 Table — (DOCX) [file pone.0246686.s004.docx]

**S2 Table.** **Percentage of respondents who selected having research conducted on cancer-related topics by demographic characteristics, including age, race/ethnicity, and geographic location.**

| **Age** | | | | | |
| --- | --- | --- | --- | --- | --- |
|  | **18-59 years** | | **60+ years** | |  |
|  | N | % | N | % | p-value ^a^ |
| Tobacco cessation | 33 | 13% | 85 | 8% | 0.09 |
| Cancer prevention | 154 | 59% | 523 | 52% | 0.06 |
| Cancer screening | 134 | 51% | 445 | 44% | 0.06 |
| Cancer treatment | 191 | 73% | 651 | 64% | **0.02** |
| Cancer survivorship | 86 | 33% | 286 | 28% | 0.24 |
| Cancer clinical trials | 147 | 56% | 544 | 54% | 0.63 |
| Nutrition and cancer | 122 | 47% | 326 | 32% | **<0.001** |
| Genetics and cancer | 127 | 49% | 404 | 40% | **0.02** |
| Biobanking and cancer | 88 | 34% | 299 | 30% | 0.31 |
| Cancer caregiving | 56 | 21% | 133 | 13% | **0.003** |
| Other | 2 | 1% | 6 | 1% | 0.75 |
| **Total Who Responded** | 261 |  | 1,013 |  |  |
| **Race/Ethnicity** | | | | | |
|  | **Hispanic or Non-White** | | **Non-Hispanic White** | |  |
| Tobacco cessation | 13 | 10% | 104 | 9% | 0.77 |
| Cancer prevention | 77 | 57% | 601 | 53% | 0.52 |
| Cancer screening | 62 | 46% | 512 | 45% | 0.94 |
| Cancer treatment | 90 | 67% | 750 | 66% | 0.89 |
| Cancer survivorship | 50 | 37% | 322 | 28% | 0.07 |
| Cancer clinical trials | 72 | 53% | 619 | 54% | 0.60 |
| Nutrition and cancer | 66 | 49% | 384 | 34% | **0.001** |
| Genetics and cancer | 59 | 44% | 475 | 42% | 0.88 |
| Biobanking and cancer | 47 | 35% | 338 | 30% | 0.36 |
| Cancer caregiving | 31 | 23% | 160 | 14% | **0.02** |
| Other | 3 | 2% | 5 | 0% | **0.01** |
| **Total Who Responded** | 135 |  | 1,136 |  |  |
| **Area** | | | | | |
|  | **Catchment Area** | | **Outside Catchment Area** | |  |
| Tobacco cessation | 86 | 9% | 32 | 9% | 0.51 |
| Cancer prevention | 490 | 54% | 187 | 52% | 0.42 |
| Cancer screening | 421 | 46% | 158 | 44% | 0.34 |
| Cancer treatment | 598 | 66% | 243 | 68% | 0.69 |
| Cancer survivorship | 264 | 29% | 109 | 30% | 0.74 |
| Cancer clinical trials | 500 | 55% | 188 | 52% | 0.28 |
| Nutrition and cancer | 315 | 35% | 132 | 37% | 0.63 |
| Genetics and cancer | 371 | 41% | 158 | 44% | 0.41 |
| Biobanking and cancer | 266 | 29% | 118 | 33% | 0.30 |
| Cancer caregiving | 139 | 15% | 51 | 14% | 0.43 |
| Other | 5 | 1% | 3 | 1% | 0.56 |
| **Total Who Responded** | 910 |  | 360 |  |  |
| ^a^ *p*-values calculated from a two-sided chi-square test  ^b^ Bolded p-values are significant at an α-level of 0.05 | | | | | |
