# Supplementary material for: Utilizing a large-scale biobanking registry to assess patient priorities and preferences for cancer research and education
Source: PLoS One. 2021 Feb 5;16(2):e0246686. doi: 10.1371/journal.pone.0246686 (PMC7864448; doi:10.1371/journal.pone.0246686)
Supplement: S3 Table — (DOCX) [file pone.0246686.s005.docx]

**S3 Table.** **Percentage of respondents who selected having research conducted on multiple cancer-related issues by demographic characteristics, including age, race/ethnicity, and geographic location.**

| **Age** | | | | | |
| --- | --- | --- | --- | --- | --- |
|  | **18-59 years** | | **60+ years** | |  |
|  | N | % | N | % | p-value ^a^ |
| Housing, transportation, childcare, job | 64 | 26% | 125 | 14% | **<0.001** |
| Insurance Issue | 117 | 48% | 297 | 32% | **<0.001** |
| Talking to my oncologist | 118 | 48% | 496 | 54% | 0.10 |
| Talking to my primary care physician | 41 | 17% | 140 | 15% | 0.64 |
| Talking to my friends/family | 26 | 11% | 81 | 9% | 0.50 |
| Caretakers of cancer patients | 59 | 24% | 189 | 20% | 0.27 |
| Emotional challenges due to cancer | 127 | 52% | 419 | 45% | 0.09 |
| Memory and concentration problems | 93 | 38% | 271 | 29% | **0.01** |
| Physical side effects of cancer | 144 | 59% | 525 | 57% | 0.66 |
| Fatigue and poor sleep | 107 | 44% | 287 | 31% | **<0.001** |
| Diet and exercise | 109 | 44% | 276 | 30% | **<0.001** |
| Fertility options after cancer | 32 | 13% | 58 | 6% | **0.001** |
| Other | 2 | 1% | 6 | 1% | 0.78 |
| **Total Who Responded** | 245 |  | 924 |  |  |
| **Race/Ethnicity** | | | | | |
|  | **Hispanic or Non-White** | | **Non-Hispanic White** | |  |
| Housing, transportation, childcare, job | 29 | 24% | 159 | 15% | **0.03** |
| Insurance Issue | 46 | 38% | 366 | 35% | 0.69 |
| Talking to my oncologist | 68 | 56% | 549 | 52% | 0.59 |
| Talking to my primary care physician | 30 | 25% | 150 | 14% | **0.01** |
| Talking to my friends/family | 20 | 17% | 88 | 8% | **0.01** |
| Caretakers of cancer patients | 32 | 26% | 216 | 21% | 0.24 |
| Emotional challenges due to cancer | 60 | 50% | 490 | 47% | 0.74 |
| Memory and concentration problems | 48 | 40% | 315 | 30% | 0.06 |
| Physical side effects of cancer | 76 | 63% | 598 | 57% | 0.34 |
| Fatigue and poor sleep | 51 | 42% | 344 | 33% | 0.07 |
| Diet and exercise | 55 | 45% | 333 | 32% | **0.01** |
| Fertility options after cancer | 14 | 12% | 75 | 7% | 0.20 |
| Other | 2 | 2% | 6 | 1% | 0.17 |
| **Total Who Responded** | 121 |  | 1,049 |  |  |
| **Area** | | | | | |
|  | **Catchment Area** | | **Outside Catchment Area** | |  |
| Housing, transportation, childcare, job | 132 | 16% | 56 | 17% | 0.93 |
| Insurance Issue | 290 | 35% | 122 | 37% | 0.73 |
| Talking to my oncologist | 442 | 53% | 172 | 51% | 0.48 |
| Talking to my primary care physician | 129 | 16% | 52 | 16% | 0.83 |
| Talking to my friends/family | 69 | 8% | 38 | 11% | 0.12 |
| Caretakers of cancer patients | 178 | 21% | 68 | 20% | 0.54 |
| Emotional challenges due to cancer | 394 | 47% | 152 | 46% | 0.44 |
| Memory and concentration problems | 258 | 31% | 103 | 31% | 0.79 |
| Physical side effects of cancer | 481 | 58% | 186 | 56% | 0.38 |
| Fatigue and poor sleep | 287 | 35% | 106 | 32% | 0.27 |
| Diet and exercise | 279 | 34% | 106 | 32% | 0.43 |
| Fertility options after cancer | 66 | 8% | 24 | 7% | 0.44 |
| Other | 5 | 1% | 3 | 1% | 0.58 |
| **Total Who Responded** | 830 |  | 334 |  |  |
| ^a^ *p*-values calculated from a two-sided chi-square test  ^b^ Bolded p-values are significant at an α-level of 0.05 | | | | | |
