# Supplementary material for: Utilizing a large-scale biobanking registry to assess patient priorities and preferences for cancer research and education
Source: PLoS One. 2021 Feb 5;16(2):e0246686. doi: 10.1371/journal.pone.0246686 (PMC7864448; doi:10.1371/journal.pone.0246686)
Supplement: S1 File — (PDF) [file pone.0246686.s006.pdf]

S1 File. Survey.

The goal of this short survey is to understand what you are interested in learning about and what areas of research are important to you. We will use the summarized data from all people who respond to develop our future Total Cancer Care eLetters and help inform our clinicians and researchers at Moffitt about what research is important to our patients. Thank you for taking time to complete this survey!

1. Please indicate your interest based on the following top cancers in our catchment area – this includes the 15 counties around Moffitt from which the majority of our patients live

**Cancer Type**	**What cancer types are you interested in learning more about? *Mark all that apply***	**What cancer types do you think should be Moffitt's highest research priority? *Mark all that apply***
Breast	☐	☐
Lung	☐	☐
Melanoma	☐	☐
Prostate	☐	☐
Colorectal	☐	☐
Bladder	☐	☐
Head and neck	☐	☐
Non-Hodgkin lymphoma	☐	☐
Kidney	☐	☐
Brain tumors	☐	☐
Thyroid	☐	☐
Uterine	☐	☐
Cervical	☐	☐
Ovarian	☐	☐
Other cancer (please specify): ________________________	☐	☐
None	☐	☐

2. Please indicate your interest based on the following cancer-related topics:

**Cancer-Related Topic**	**What cancer-related topics are you interested in learning more about? *Mark all that apply***	**What cancer-related topics do you think should be Moffitt's highest research priority? *Mark all that apply***
Tobacco cessation	☐	☐
Cancer prevention	☐	☐
Cancer screening	☐	☐

Cancer treatment	☐	☐
Cancer survivorship	☐	☐
Cancer clinical trials	☐	☐
Nutrition and cancer	☐	☐
Genetics and cancer	☐	☐
Biobanking (collecting human tissue for research) and cancer	☐	☐
Cancer caregiving	☐	☐
Other cancer-related topics (please specify): ______________________________	☐	☐
None	☐	☐

3. Please indicate your interest based on the following issues often faced by cancer patients:

**Cancer-Related Issues**	**What cancer-related issues are you interested in learning more about?** ***Mark all that apply***	**What cancer-related issues do you think should be Moffitt's highest research priority?** ***Mark all that apply***
Housing, transportation, child care, and job concerns	☐	☐
Insurance issues	☐	☐
Talking to my oncology provider (e.g., new treatment options, understanding test results)	☐	☐
Talking to my primary care physician	☐	☐
Talking to my family and friends	☐	☐
Care-takers of cancer patients	☐	☐
Emotional challenges due to cancer (e.g., anxiety)	☐	☐
Memory and concentration problems	☐	☐
Physical side effects of cancer (e.g., weight change, pain, nausea, hot flashes)	☐	☐
Fatigue and poor sleep	☐	☐
Diet and exercise	☐	☐
Fertility options after cancer	☐	☐
Other cancer-related issues (please specify): ______________________________	☐	☐

None	☐	☐

4. How would you like to receive cancer education/information from Moffitt? *Mark all that apply*
   - ☐ Total Cancer Care eLetter
   - ☐ Events in my community (e.g., health fairs, education workshops)
   - ☐ Mailed materials about Moffitt
   - ☐ Emailed materials about Moffitt
   - ☐ Web-based resources
   - ☐ Social media
   - ☐ Other, please specify ____
   - ☐ I do not wish to receive cancer education/information from Moffitt
     (NOTE: This survey will not opt you out from receiving information from Moffitt)

Demographics

5. What is your age?
   - ☐ 18-24
   - ☐ 25-29
   - ☐ 30-34
   - ☐ 35-39
   - ☐ 40-44
   - ☐ 45-49
   - ☐ 50-54
   - ☐ 55-59
   - ☐ 60-64
   - ☐ 65-69
   - ☐ 70-74
   - ☐ 75-79
   - ☐ 80 or over

6. What is your gender?
   - ☐ Female
   - ☐ Male
   - ☐ Prefer not to say
   - ☐ Other, please specify _____________________________

7. What is your ethnicity?
   - ☐ Hispanic or Latino
   - ☐ Not Hispanic or Latino

8. What is your race? (Please select all that apply)
   - ☐ American Indian or Alaska Native
   - ☐ Asian
   - ☐ Black or African American
   - ☐ Native Hawaiian or Other Pacific Islander

- ☐ White
- ☐ Prefer not to say
- ☐ Other, please specify ______________________________

9. What is your county of residence?
   - ☐ Charlotte
   - ☐ Citrus
   - ☐ DeSoto
   - ☐ Hardee
   - ☐ Hernando
   - ☐ Highlands
   - ☐ Hillsborough
   - ☐ Lake
   - ☐ Lee
   - ☐ Manatee
   - ☐ Pasco
   - ☐ Pinellas
   - ☐ Polk
   - ☐ Sarasota
   - ☐ Sumter
   - ☐ Other Florida county, please specify ______________________________
   - ☐ Out of state, please specify state or country______________________________
